# Detection of Rib Metastases in Patients with Lung Cancer: A Comparative Study of MRI, CT and Bone Scintigraphy

**DOI:** 10.1371/journal.pone.0052213

**Published:** 2012-12-27

**Authors:** Yan-Qing Chen, Yang Yang, Yan-Fen Xing, Sen Jiang, Xi-Wen Sun

**Affiliations:** 1 Soochow University School of Medicine, Suzhou, Jiangsu, China; 2 Department of Radiology, Shanghai Pulmonary Hospital, Tongji University School of Medicine, Shanghai, China; University of Porto, Portugal

## Abstract

We retrospectively investigated the imaging findings of bone scintigraphy, chest CT and chest MRI in 55 cases of lung cancer. The sensitivity, specificity and accuracy of the detection of rib metastases were compared between imaging modalities on both a per-lesion and a per-patient basis. On a per-lesion basis, MRI sensitivity and accuracy were significantly higher than that of bone scintigraphy and CT (P<0.05). The sensitivities, specificities, and accuracy levels between CT and bone scintigraphy did not differ on either a per-lesion or per-patient basis (P>0.05). MRI appears to be superior for the detection of ribs metastases in lung cancer.

## Introduction

Lung cancer is the most common cause of cancer-related death among men and women worldwide [1], [2]. The spine is the most common site of bone deposits, with ribs being the second most common site [3], [4]. The early and accurate diagnosis of rib metastases in lung cancer is therefore essential to provide optimal patient management.

Currently, bone scintigraphy and/or CT scanning are commonly used for the detection of rib metastases in patients with lung cancer, with the occasional use of MRI, and rarely, PET/CT scanning [5]. However, no direct comparison of the effectiveness of CT, bone scintigraphy and MRI in the assessment of rib metastases in lung cancer patients has been reported to date. Therefore, we retrospectively compared these three modalities for the assessment of rib metastases in a patient population with lung cancer.

## Materials and Methods

### Subjects

Patients were assessed for inclusion who had been referred to our hospital with a histopathological diagnosis of lung cancer and who had undergone bone scintigraphy, CT scan or MRI imaging within the previous 4 weeks [6], [7]. A total of 55 patients were enrolled (42 males, 13 females). The mean age was 58 years (range, 31 to 77 years). There were 42 patients (76.4%) older than 50 years of age. The histopathological diagnoses were as follows: squamous cell carcinoma (n = 19, 34.5%); adenocarcinoma (n = 26, 47.3%); adenosquamous carcinoma (n = 3, 5.5%); combined small cell carcinoma (n = 5, 9.1%); and non-small cell carcinoma alone (n = 2, 3.6%).

### Imaging Techniques

All patients underwent radionuclide bone scintigraphy using a SPECT scanner (e-CAM; Siemens Medical Solution, Forchheim, Germany). Scintigraphy was performed with dual-head detectors equipped with a low-energy, high-resolution collimator. All patients underwent whole-body bone scanning using ^99m^Tc, matrix = 256×1024.

Thoracic CT scanning was performed with a 64-section CT scanner (Siemens, Germany) or a 40-section CT scanner (Philips, The Netherlands). Acquisition parameters were the same between both scanner types, as follows: 150–200 mA, 120 kVp, matrix = 512×512, reconstructed section thickness = 5 mm and 1 mm. Unless otherwise specified, the section thickness used was 5 mm for the retrospective detection of rib metastases. On the basis of the detection of lesions at 5 mm, an analysis at 1 mm was undertaken.

Thoracic MR imaging was performed with a 1.5T system (Archiva, Philips, the Netherlands). The imaging sequence and parameters were as follows: T1 weighted imaging (T1WI), Gd-DTPA enhanced T1WI imaging, T2WI imaging with fat suppression; matrix = 512×512, section thickness = 5 mm, axial scanning imaging.

### Imaging Analysis

Images from the three imaging modalities were analyzed independently by six different radiologists. Bone scintigraphy was assessed by two radionuclide radiologists, CT images were read by two radiologists with expertise in the field, and MR images were read by two radiologists with expertise in the interpretation of MRI. Each radiologist had at least 10 years’ of experience in their respective areas. The radiologists were unaware of the clinical and biological findings or of any other imaging studies relevant to the individual patient. They were aware, however, that all patients had lung cancer and were being evaluated for the presence of rib metastases. The six readers scored the number and volume of rib metastases depicted at each examination.

On MRI, a rib lesion was considered to be suspicious of a metastasis if it had a slightly high signal, high signal or mixed signal on T2WI imaging with fat suppression, or a slightly low signal, low signal, isointense signal or a mixed signal on T1WI that enhanced slightly, obviously or heterogeneously on Gd-DTPA enhanced T1WI imaging. Areas of abnormal uptake of ^99m^Tc-MDP on bone scintigraphy that could not be definitively diagnosed as benign were considered as suspicious of metastases. A rib lesion with an abnormal density, with or without an abnormal shape or a soft tissue mass on CT, which could not be diagnosed as benign with any certainty, was considered suspicious of a metastasis.

The final diagnosis of a rib metastasis was determined based on the results of initial and follow-up bone scintigraphy, CT, and MRI examinations and pathological examinations. The lesions that were suspected of being metastases based on the initial examinations were confirmed as metastases when the tissues were pathologically proved to be metastatic or the lesions became larger during the follow-up periods or decreased in size after treatment. The final diagnosis of rib metastasis was arrived at by consensus at a conference attended by the radiologists, pathologists and clinicians.

### Statistical Analysis

To compare the diagnostic capability on a per-lesion basis and on a per-patient basis, the χ^2^ test was used to compare the sensitivity, specificity, and accuracy of bone scintigraphy, CT and MRI. A P-value less than 0.05 was considered statistically significant for all analyses. Statistical analysis was conducted with SPSS 17.0 statistical software (SPSS, Chicago, IL).

## Results

Of the 55 patients, 30 had a total of 120 rib metastases (Tables 1, 2), which were proven on clinical grounds, imaging and imaging follow-up (follow-up period, 3–18 months). Analysis on a per-patient basis showed that there was no significant difference in the sensitivity, specificity or accuracy among CT, bone scintigraphy and MRI imaging (P>0.05, Table 1). When the data were analyzed on a per-lesion basis, the sensitivity and accuracy of MRI for the detection of rib metastases were significantly higher than that of bone scintigraphy and CT (P<0.05, Table 2). There was no significant difference in the sensitivity, specificity, and accuracy between CT and bone scintigraphy (P>0.05, Table 2).

**Table 1:** Number of bone metastases identified on imaging (per patient, n = 55).

	Bone scintigraphy	CT	MRI	*P*-value
True positive	28	27	30	
True negative	21	20	25	
False positive	4	5	0	
False negative	2	3	0	
Sensitivity (%)	93.3	90.0	100.0	0.959
Specificity (%)	84.0	80.0	100.0	0.848
Accuracy (%)	89.1	85.5	100.0	0.838

Not significant, P>0.05; χ^2^ test.

**Table 2:** Number of bone metastases identified on imaging (per lesion, n = 135).

	Bone scintigraphy	CT	MRI	*P*-value
True positive	62	65	120	
True negative	9	7	14	
False positive	6	8	1	
False negative	58	55	0	
Sensitivity* (%)	51.7	54.2	100.0	0.001
Specificity (%)	60.0	46.7	93.3	0.471
Accuracy** (%)	52.6	53.3	99.3	0.000

* Significant difference between CT, bone scintigraphy and MRI (P = 0.001, χ^2^ test).

* Significant difference between CT and MRI (P = 0.002, χ^2^ test).

* Significant difference between bone scintigraphy and MRI (P = 0.001, χ^2^ test).

** Significant difference between CT, bone scintigraphy and MRI (P = 0.000, χ^2^ test).

** Significant difference between CT and MRI (P = 0.001, χ^2^ test).

** Significant difference between bone scintigraphy and MRI (P = 0.001, χ^2^ test).

### Detection of Rib Metastases with Bone Scintigraphy

Twenty-eight patients with rib metastases and 62 metastatic rib lesions were correctly identified as true-positive findings by bone scintigraphy. On bone scintigraphy, all 62 true-positive metastatic rib lesions demonstrated uptake. Solitary metastases were present in 12 patients and 16 patients had multiple lesions.

### Detection of Rib Metastases with CT Imaging

When an observation section thickness of 5 mm was used, a total of 27 patients with rib metastases and a total of 65 metastatic rib lesions were correctly identified as true-positives by CT. There were eight patients with solitary metastases and 19 with multiple metastases. CT scans depicted osteoblastic changes in eight patients (16 osteoblastic metastatic lesions), osteolytic changes in seven (16 osteolytic metastatic lesions), and mixed changes in 12 (12 osteoblastic lesions, 11 osteolytic lesions, and 10 mixed metastatic lesions) (Table 3, 4). CT scans detected predominantly mixed changes on a per-patient basis (44.5%, 12/27), with osteoblastic changes the next most common type (29.6, 8/27). However, CT scans depicted predominantly osteoblastic (43.1%, 28/65) and osteolytic changes (41.5%, 27/65) on a per-lesion basis.

**Table 3:** Distribution on a per-patient basis of different types of rib metastases in lung cancer, comparing 5 mm slice thickness and 1 mm slice thickness.

Metastases type	5 mm slice thickness	1 mm slice thickness
Osteoblastic (n, %)	8 (29.6)	1 (3.7)
Osteolytic (n, %)	7 (25.9)	2 (7.4)
Mixed (n, %)	12 (44.5)	24 (88.9)
Total	27 (100.0)	27 (100.0)

**Table 4:** Distribution on a per-lesion basis of different types of rib metastases in lung cancer, comparing 5 mm slice thickness and 1 mm slice thickness.

Metastases type	5 mm slice thickness	1 mm slice thickness
Osteoblastic (n, %)	28 (43.1)	5 (7.7)
Osteolytic (n, %)	27 (41.5)	12 (18.5)
Mixed (n, %)	10 (15.4)	48 (73.8)
Total	65 (100.0)	65 (100.0)

On the basis of the detection of lesions at 5 mm, an analysis at 1 mm was undertaken. CT scans depicted osteoblastic changes in one patient (1 osteoblastic lesion), osteolytic changes in two patients (3 osteolytic lesions), and mixed changes in 24 (4 osteoblastic lesions, 9 osteolytic and 48 mixed lesions) (Table 3, 4). CT scans revealed mostly mixed changes, both on a per-patient basis (88.9%, 24/27) and on a per-lesion basis (73.8%, 48/65). The per-patient and per-lesion comparisons of the distribution of different types of rib metastases in lung cancer between 5 mm slice thickness and 1 mm slice thickness can be seen in Table 3 and Table 4, respectively.

### Detection of Rib Metastases with MRI

Thirty patients with rib metastases and 120 metastatic rib lesions were correctly identified as true-positive findings by MRI. All 30 patients had multiple lesions.

## Discussion

On a per-patient basis, CT scans detected predominantly mixed changes (44.5%) and osteoblastic changes (29.6%), which differed from the results of most similar studies that have mainly reported osteolytic changes on CT imaging (range, 73.1% to 89.7%) [8]. However, most research on bone metastases has focused on the whole body or the axial skeleton, whereas we studied the ribs alone. The microenvironments of different bone sites are unique, and the type of interactions between the bone microenvironment and tumor cells can vary, which can potentially give rise to osteolytic (bone resorption), osteoblastic (bone-forming) or mixed metastases [9].

On a per-lesion basis, CT scans in our study depicted predominantly osteoblastic (43.1%) and osteolytic changes (41.5%). Our study was on both a per-lesion basis and a per-patient basis. However, most studies on bone metastases, especially the type of rib metastases in lung cancer, have been on a per-patient basis. As a result, it is difficult to compare these studies.

**Figure 1:**
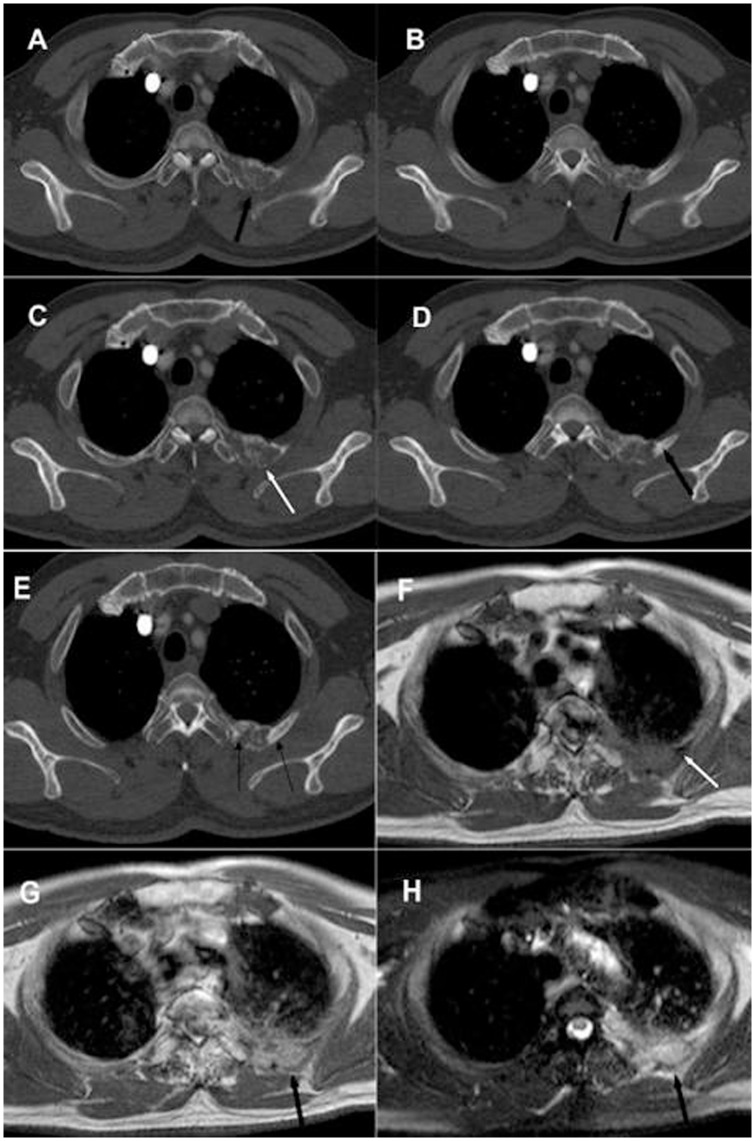
The imaging manifestations of rib metastases in lung cancer, comparing 5 mm and 1 mm slice thickness on CT, and MRI. A, B. Two consecutive 5 mm levels of CT images showing a rib metastasis (black arrow); CT depicted predominantly osteolytic and expansive changes, osteoblastic changes were not obvious. C, D, E. Thickness of 1 mm interception of three discontinuous CT dimensions showing typical mixed changes, osteoblastic changes (black arrow), and osteolytic changes (white arrow). F. T1WI shows an area of isometric and low-mixed signal intensity; the area that showed osteoblastic changes on CT reveals low signal intensity on T1WI (white arrow). G. The lesion enhances heterogeneously on Gd-DTPA enhanced T1WI imaging (black arrow). H. T2WI (FS) shows high, slightly high, isometric and low-mixed signal intensity (black arrow).

**Figure 2:**
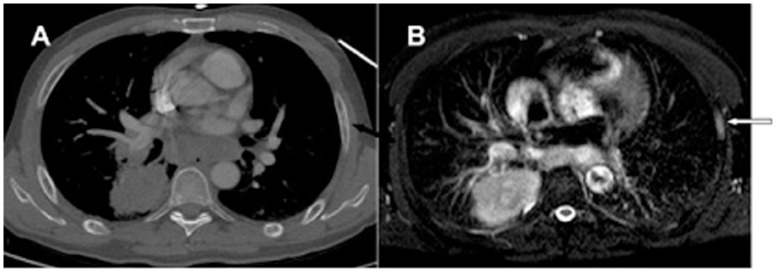
Minimal osteolytic changes: the initial manifestations of bone metastases and the primary stage of osteoblastic metastases. A. CT image showing a rib metastasis on the left, presenting as an intramedullary small patchy hyperdensity and a peripheral hypodense spot (black arrow). B. The lesion appears as an intramedullary area of slightly high signal on T2WI (FS) (white arrow).

When the reconstructed section thickness was 5 mm, CT images predominantly depicted mixed changes (44.5%) on a per-patient basis, and predominantly osteoblastic changes (43.1%) and osteolytic changes (41.5%) on a per-lesion basis. However, when the reconstructed section thickness was 1 mm, CT scans revealed mostly mixed changes (Figure 1), on both a per-patient basis (88.9%) and per-lesion basis (73.8%). These results validate previous results that can partially explain the hypothesis that the activation of osteoclasts is the precondition for bone metastasis, and that every metastatic bone lesion commences with osteolysis [10], [11]. As such, the initial manifestations of bone metastases are minimal osteolytic changes, which are essentially the primary stage of osteolytic, mixed and osteoblastic metastases (Figure 2). This is both a microscopic and a macroscopic process. The traditional understanding of rib metastases on CT is now inappropriate as advances in imaging technology have shown that these changes represent early, mid- or late stages of metastasis.

There were no significant differences in the sensitivity, specificity and accuracy of CT and bone scintigraphy images (P>0.05), which differed from one previous study [12] but was consistent with one other [13]. However, as mentioned, these studies examined the whole body or the axial skeleton. Our focus on the ribs means it is difficult to directly compare these studies.

**Figure 3:**
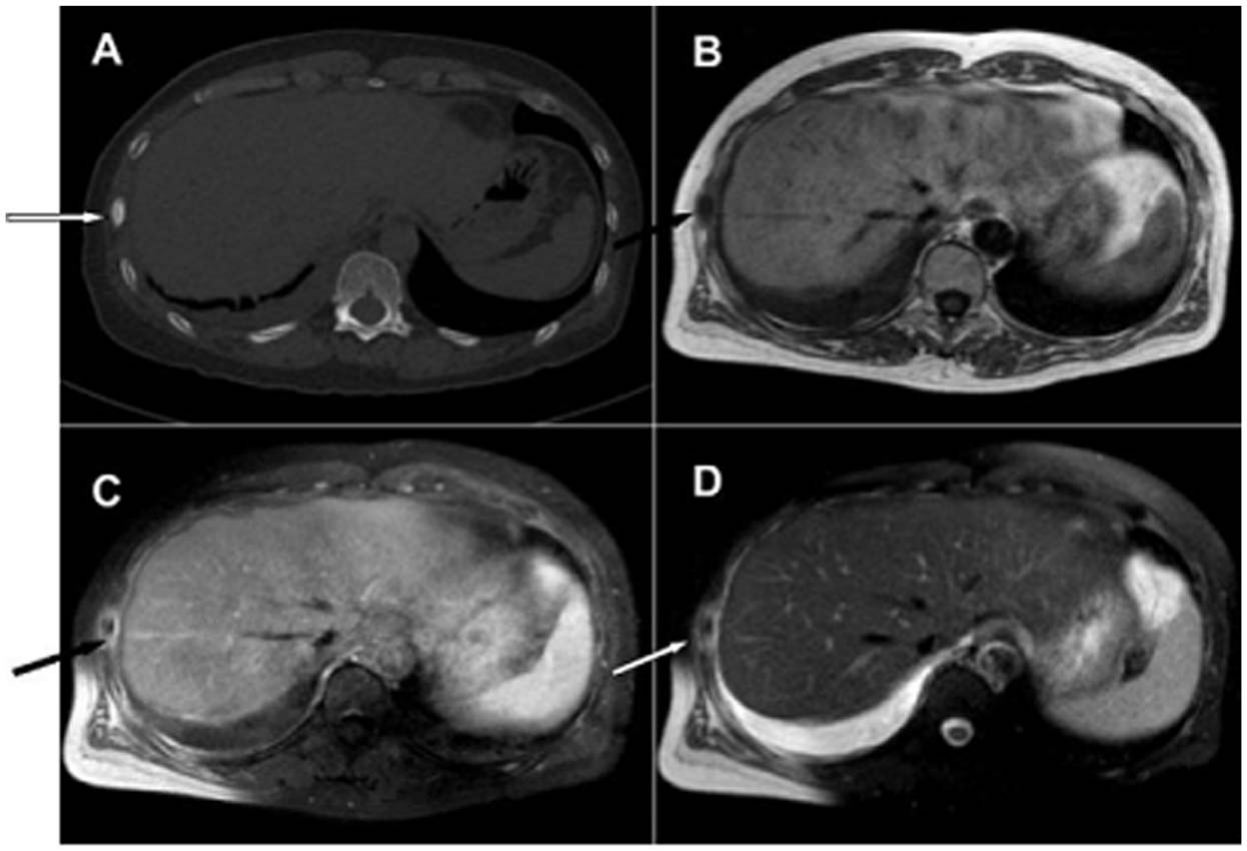
MRI in the detection and extent of osteoblastic metastatic lesions through direct and indirect signs. A. CT image showing an osteoblastic metastatic lesion of a right rib, presenting as an intramedullary homogeneously high density (white arrow). B. T1WI showing an area of low signal intensity (black arrow). C. The lesion enhances slightly and heterogeneously on Gd-DTPA enhanced T1WI imaging with peripheral edema (black arrow). D. T2WI (FS) showed intramedullary areas of high, slightly high, isometric and low-mixed signal intensity with peripheral edema (white arrow).

In our study, the sensitivity of MRI for osteoblastic metastatic lesions was higher than bone scintigraphy and CT. Osteoblastic metastatic lesions, which were detected with CT and/or bone scintigraphy, were all detected with MRI. In addition, MRI may be more sensitive and accurate in the detection and extent of bone lesions through indirect signs, such as peripheral inflammatory reactions, edema and the presence of a soft tissue swelling or mass (Figure 3). These results differ from previous studies in this field, which have reported a lower sensitivity for MRI in the detection of osteoblastic metastatic lesions, especially in small bones such as the ribs [6].

The activation of osteoclasts is a precondition for bone metastases, and every metastatic bone lesion begins with osteolysis [10]. Bone loss occurs with increasing age in both men and women, reaching 0.3% and 0.5% per year, respectively, once peak bone mass has been achieved. Such bone loss results in the increased porosity in cortical and trabecular bones, and the progression of general osteoporosis accelerates with age [14]. In our study, there were 42 patients (76.4%) above 50 years of age and the mean age was 58 years. Osteoporosis in this population was obvious on imaging. In addition, cancer can induce local osteoporosis [15]. All of these factors can affect the detection and differential diagnosis of early-stage rib metastatic lesions, which mainly manifest as spots or nodules, as small patchy and poorly characterized low density shadows in the rib bone marrow on CT. The sensitivity and specificity of bone scintigraphy is also low for early small lesions [16]. The sensitivity of MRI was significantly higher than bone scintigraphy and CT in our study, especially for small osteolytic lesions. The sensitivity, specificity and accuracy of MRI in the differential diagnosis between small osteolytic metastatic lesions and osteoporosis was relatively high, and these results are consistent with those of Baur-Melnyk and Reiser [17], which focused on bone metastases in large bones in particular. Another study [18] showed that the sensitivity of MRI for bone marrow changes was significantly higher than that of bone scintigraphy. MRI can detect lesions when cancer cells have just infiltrated the bone trabecular, before obvious osteolytic or osteoblastic changes appear. In current clinical practice, the use of preoperative imaging to exclude bone metastases in lung cancer mainly depends on bone scintigraphy and/or CT scanning, which could potentially result in missed diagnoses and affect the selection of treatment protocols and prognostication.

At present, some suggest that we should consider surgical treatment for solitary bone metastases from lung cancer, especially solitary rib metastasis in non-small cell lung cancer, as its prognosis tends to be better than that achieved with non-surgical management [19]. In our study, a solitary rib metastasis was detected by CT in eight cases and by bone scintigraphy in 12, but all 30 cases were shown to have multiple rib metastases on MRI. This suggests that bone scintigraphy or CT scans for the preoperative screening of bone lesions is inappropriate and potentially inaccurate. The limitations of available imaging techniques may undermine the extent of bone lesions. The underlying mechanisms of bone metastases, including hematogenous or lymphatic spread, and the implantation of metastases makes it difficult to understand the occurrence of a solitary lesion, although it is possible that there are complex mechanisms that can cause solitary bone metastases.

The sample size of 55 cases (30 cases with definite metastases) in our study was small, and therefore the results potentially lack adequate statistical significance. Further studies with larger numbers are needed to validate our findings. The other limitations of our study include the selection bias as a consequence of the retrospective design of our study. Currently, bone scintigraphy and/or CT are commonly used for the detection of rib metastases in patients with lung cancer, with the occasional use of MRI. Hence, patients without a clinical suspicion of metastatic disease, in addition to those where the presence of metastases on bone scintigraphy and/or CT scanning was uncertain, may not have been included.
